# Hydroxytyrosol as a Mitochondrial Homeostasis Regulator: Implications in Metabolic Syndrome and Related Diseases

**DOI:** 10.3390/antiox14040398

**Published:** 2025-03-27

**Authors:** Jie Xu, Huanglong Wei, Zhenyu Sun, Wankang Li, Jiangang Long, Jiankang Liu, Zhihui Feng, Ke Cao

**Affiliations:** 1 Center for Mitochondrial Biology and Medicine, The Key Laboratory of Biomedical Information Engineering of Ministry of Education, School of Life Science and Technology, Xi’an Jiaotong University, Xi’an 710049, China; xj0322@xjtu.edu.cn (J.X.); weihuanglong@stu.xjtu.edu.cn (H.W.); zhenyu_sun@stu.xjtu.edu.cn (Z.S.); lwk0626@stu.xjtu.edu.cn (W.L.); jglong@mail.xjtu.edu.cn (J.L.); jkliu@uor.edu.cn (J.L.); 2 School of Health and Life Sciences, University of Health and Rehabilitation Sciences, Qingdao 266071, China; 3 Frontier Institute of Science and Technology, Xi’an Jiaotong University, Xi’an 710049, China

**Keywords:** hydroxytyrosol, mitochondria, metabolic syndrome, mitochondrial delivery system, synergistic therapies

## Abstract

Hydroxytyrosol (HT), a principal bioactive phytochemical abundant in Mediterranean dietary sources, has emerged as a molecule of significant scientific interest owing to its multifaceted health-promoting properties. Accumulating evidence suggests that HT’s therapeutic potential in metabolic disorders extends beyond conventional antioxidant capacity to encompass mitochondrial regulatory networks. This review synthesizes contemporary evidence from our systematic investigations and the existing literature to delineate HT’s comprehensive modulatory effects on mitochondrial homeostasis. We systematically summarized the impact of HT on mitochondrial dynamics (fusion/fission equilibrium), biogenesis and energy metabolism, mitophagy, inter-organellar communication with the endoplasmic reticulum, and microbiota–mitochondria crosstalk. Through this multidimensional analysis, we established HT as a mitochondrial homeostasis modulator with potential therapeutic applications in metabolic syndrome (MetS) and its related pathologies including type 2 diabetes mellitus, obesity-related metabolic dysfunction, dyslipidemia, non-alcoholic steatohepatitis, and hypertension-related complications. Moreover, we further discussed translational challenges in HT research, emphasizing the imperative for direct target identification, mitochondrial-targeted delivery system development, and combinatorial therapeutic strategies. Collectively, this review provides a mechanistic framework for advancing HT research and accelerating its clinical implementation in MetS and its related diseases.

## 1. Introduction

Metabolic syndrome (MetS), a multifactorial clinical entity characterized by concurrent hyperglycemia, dyslipidemia, and hypertension, represents a global health challenge driven by escalating urbanization and lifestyle transitions. This cluster of interrelated conditions markedly amplifies susceptibility to major non-communicable diseases, including type 2 diabetes mellitus (T2DM), non-alcoholic fatty liver disease (NAFLD), and atherosclerotic cardiovascular disorders [1,2,3]. As socioeconomic development advances and lifestyles undergo transformations, the prevalence of MetS has been steadily rising, thereby imposing a substantial burden on healthcare systems and emerging as a critical public health challenge worldwide [4,5]. While insulin resistance remains the cornerstone of MetS pathophysiology [6], emerging evidence has implicated disturbances in mitochondrial homeostasis as a critical mediator of impaired insulin signaling and systemic metabolic dysregulation [7,8,9,10]. Research indicated that perturbations in mitochondrial dynamics—specifically unbalanced fusion–fission equilibria—compromised cellular bioenergetics and redox balance, fostering insulin resistance and metabolic disorders in metabolically active tissues [7]. Additionally, concomitant reductions in mitochondrial biogenesis [8] and defective mitophagy [10] may impair oxidative phosphorylation (OXPHOS) efficiency and further exacerbate energy metabolism imbalances, promoting the onset of insulin resistance and driving the manifestation of MetS. Intriguingly, recent insights have revealed that interorganelle crosstalk, particularly mitochondria–endoplasmic reticulum (ER) interactions [11,12] and mitochondria–gut microbiota axis [13], are capable of modulating mitochondrial networks, suggesting novel axes in MetS pathogenesis. Collectively, these findings position mitochondrial homeostasis as a strategic therapeutic target for mitigating MetS progression and its complications.

**Figure 1:**
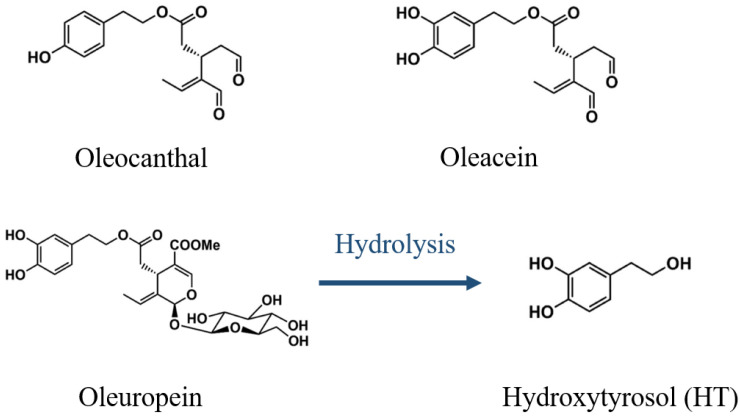
The molecular structures of oleocanthal, oleacein, oleuropein, and hydroxytyrosol (HT). Notably, HT is a hydrolysate derived from oleuropein.

The Mediterranean diet, which has garnered considerable attention for its beneficial impacts on human health, derives much of its efficacy from olive oil polyphenols [14,15]. The key polyphenolic compounds identified in olive oil include oleocanthal, oleacein, and oleuropein, while hydroxytyrosol (HT) is a metabolite of oleuropein [16] (as illustrated in Figure 1). Plant-derived metabolites, especially polyphenols, significantly contribute to stress antagonism in plant through combating exogenous stressors and pathogenic invasions, a phenomenon attributed to their dual capacity for free radical scavenging and mitochondrial homeostasis regulation [17,18,19,20]. Although direct evidence regarding HT’s regulatory role in plant stress responses remains undocumented, it is reasonable to speculate the critical involvement of HT in an olive’s stress adaptation mechanisms because of its antioxidant, anti-viral, and antibacterial properties (Table 1). Beyond its well-documented antioxidant properties, which have been consolidated by us [21,22,23] and others [24,25,26], our laboratory [27,28,29], in conjunction with other research groups [30,31,32,33,34], has demonstrated the pleiotropic effects of HT in various metabolic diseases, including obesity [27,30], T2DM [28,31], NAFLD [28,32], and cardiovascular disorders [29,33,34]. It is noteworthy to emphasize that recent evidence has suggested that the beneficial effects of HT in these metabolic-related conditions may not solely stem from its antioxidant properties, but rather through its capacity to modulate signaling pathways integral to mitochondrial homeostasis [27,28,29,35,36]. Considering mitochondrial homeostasis disruptions as pivotal drivers of MS and associated diseases, HT could function as a modulator within this network, potentially alleviating the onset of MS and related conditions.

This review synthesizes contemporary insights into HT’s mitochondrial-targeted mechanisms and their implications for MetS management. We critically evaluated HT’s roles in (1) balancing mitochondrial fusion/fission dynamics, (2) enhancing oxidative metabolism and biogenesis, (3) restoring mitophagic flux, and (4) modulating mitochondria–ER interactions and gut microbiota–mitochondria crosstalk. Furthermore, we discuss translational challenges in harnessing HT’s therapeutic potential, including the identification of precise downstream targets of HT, research into mitochondrial-targeted delivery systems, and the investigation of synergistic therapies combining HT with other compounds. By delineating HT’s multifaceted actions within the mitochondrial homeostasis network, this review aimed to lay the foundation for further elucidating the pharmacological mechanisms of HT and advancing its clinical applications.

## 2. The Regulatory Mechanisms of Hydroxytyrosol on Mitochondrial Homeostasis

### 2.1. Modulation of Hydroxytyrosol on Mitochondrial Fusion and Fission

Mitochondria exhibit adaptive morphological variations across different tissues, governed by the intricate processes of fusion and fission [42]. The equilibrium between mitochondrial fusion and fission is critical for maintaining cellular mitochondrial homeostasis [43]. Mitochondrial fusion is regulated by mitofusin 1 (Mfn1), Mfn2, and optic atrophy protein 1 (OPA1). Mfn1 and Mfn2 are both dynein-related GTPases. Fusion is essential for mitochondrial DNA stability, respiration, membrane potential, and calcium homeostasis. Mitochondrial fission is primarily driven by dynein-related protein 1 (DRP1), which translocates to the outer mitochondrial membrane and interacts with MFF, MID49, MID51, and FIS1 to initiate fission. This process is essential for mitochondrial quality control through coordinating with mitophagy and ensures the distribution of mitochondria during cell division. Fission is associated with a decrease in respiration, membrane potential, and ATP synthesis [44]. Emerging scientific evidence has highlighted the capacity of HT to modulate these mitochondrial dynamic events through distinct molecular pathways. Cai and colleagues [45] demonstrated that HT stabilized mitochondrial architecture by activating the AKT-GSK3β axis, thereby modulating proteolytic processing of OPA1—a critical mediator of mitochondrial fusion. Intriguingly, while low-dose HT supplementation (20 mg/kg/day) enhanced mitochondrial supercomplex assembly during exercise via dynamic remodeling, higher doses (300 mg/kg/day) paradoxically suppressed OPA1 isoform expression and supercomplex formation [46]. In line with these findings, our prior study uncovered that the acetate derivative of HT-inhibited OPA1 cleavage to mitigate oxidative stress-triggered mitochondrial dysfunction in skeletal muscle [36]. Subsequently, our laboratory’s series of work [28,29,47] has further emphasized HT’s potential on modulating mitochondrial dynamics. Specifically, we have demonstrated HT’s ability to normalize aberrant expression of Drp1 and Mfn2, counteracting lipid metabolic dysregulation in obese murine liver and muscle tissues [28]. Furthermore, HT exhibited promise in attenuating skeletal muscle atrophy [47] and pathological cardiac hypertrophy [29] resulting from exhaustive exercise. These observations collectively hint at OPA1, Drp1, and Mfn2 serving as potential therapeutic targets for HT. However, mechanistic details governing HT’s dose-dependent effects on mitochondrial dynamics warrant further exploration. Interestingly, both plant and animal mitochondria undergo fusion and fission to adapt to stress. For example, in *Arabidopsis*, cardiolipin within mitochondria regulates mitochondrial dynamics by stabilizing dynamin-related protein 3 (DRP3), the primary mitochondrial fission factor [48]. Consequently, this process plays a crucial role in the plant’s resistance to stress induced by heat and prolonged darkness [48], mirroring the animal response where Mfn2, OPA1, and Drp1 stabilize mitochondrial dynamic networks during metabolic stress [28,29,47]. This shared mechanism highlights evolutionary conservation in stress adaptation. Given HT’s regulatory role in mitochondrial dynamics in mammalian cells, it is plausible that HT may also play a significant role in stress resistance in olives by modulating the processes of mitochondrial fusion and fission.

### 2.2. Modulation of Hydroxytyrosol on Mitochondrial Biogenesis and Oxidative Phosphorylation

Mitochondria are highly dynamic organelles, which adaptively modulate their quantity and functionality in response to cellular stress [49]. A key role of this regulation is a peroxisome proliferator-activated receptor γ coactivator 1-alpha (PGC-1α), a master nuclear coactivator that regulates mitochondrial biogenesis and enhances respiratory capacity through various mechanisms. PGC-1α enhances mitochondrial uncoupling via induction of uncoupling protein 2 (UCP-2) and coordinates nuclear–mitochondrial crosstalk by regulating nuclear respiratory factors (NRFs) [50]. Specifically, PGC-1α exerts dual regulatory roles: (1) potently upregulating *NRF-1* and *NRF-2* gene expression, and (2) physically interacting with NRF-1 to coactivate mitochondrial transcription factor A (mtTFA)—a critical driver of mitochondrial DNA replication and transcription. This hierarchical regulation establishes PGC-1α as a pivotal integrator of mitochondrial adaptation under stress conditions [51]. A pivotal discovery made over a span of more than ten years in our laboratory was the identification of HT as a potent inducer of mitochondrial biogenesis in vitro [27,52,53]. Our initial observations demonstrated that low-dose HT (0.1–10 μM) augmented mitochondrial biogenesis and functional capacity in 3T3-L1 adipocytes via upregulating PGC-1α expression, increasing mitochondrial DNA (mtDNA) copy number and enhancing OXPHOS complexes I, II, III, and V activity [27]. Mechanistically, siRNA-mediated PGC-1α silencing significantly attenuated HT-induced mitochondrial complex I expression and mtDNA copy number amplification, confirming its pivotal role in HT’s bioactivity [27].

Nuclear factor E2-related factor 2 (Nrf2) is a transcription factor that responds to stress in cellular redox balance. Under non-stress conditions, Nrf2 is held in the cytoplasm by Keap1 and tagged for degradation by the Cul3 ubiquitin ligase. However, during oxidative stress, Nrf2 separates from Keap1 and moves into the nucleus, where it activates a range of protective genes that support the cell’s antioxidant defense system [54]. Notably, there is a reciprocal crosstalk between the Nrf2-regulated antioxidant pathway and the PGC1α-modulated mitochondrial biogenesis pathway. On one hand, disruptions in mitochondrial biogenesis can result in elevated production of reactive oxygen species (ROS), which subsequently activates the Nrf2 antioxidant system. On the other hand, activation of Nrf2 can contribute to the maintenance of mitochondrial biogenesis and mitochondrial function by alleviating mitochondrial oxidative damage [55,56]. Our subsequent investigations revealed HT’s dual activation of PGC-1α-driven biogenesis and Nrf2-mediated antioxidant defenses in ARPE cells, effectively countering acrolein-induced oxidative insult and OXPHOS deficits [52,53] and defending tert-butyl hydroperoxide-triggered glutathione decrease and mitochondrial dysfunction [23]. In this process, 50 μM HT pretreatment enhanced the nuclear translocation of Nrf2 and the expression of Nrf2 target genes such as *HO-1*, *NQO-1*, thereby activating phase II enzymes, which contributes to enhanced antioxidant defense [21].

We further validated HT’s benefits on mitochondrial biogenesis and OXPHOS in vivo. Specifically, HT restored PGC-1α and complex I/II expression and recovered mtDNA copy number in skeletal muscles [47] and myocardia [29] of rats after excessive exercise. Furthermore, our investigation into prenatal restraint stress rats unveiled that maternal HT supplementation significantly rescued the expression profiles of mitochondrial OXPHOS complexes and mtDNA copy number in the offspring’s hippocampus, leading to an improvement in cognitive outcomes [57]. Additionally, our experimental findings in db/db mice further demonstrated that an 8-week HT intervention effectively restored expression levels of AMPK, Sirt1, and PGC-1α—core regulators of mitochondrial biogenesis—while concurrently enhancing mitochondrial complex I activity, ultimately alleviating cerebral oxidative stress and rescuing mitochondrial dysfunction [58].

In harmony with our findings, parallel research from other groups has also underscored HT’s regulatory role in mitochondrial biogenesis and OXPHOS. Dong et al. [59] linked AMPK/PGC-1α activation to HT-induced hepatic lipid reduction in fish through coordinated mitochondrial biogenesis and autophagy. Another study suggested PKA/CREB phosphorylation as an upstream mechanism for HT-mediated PGC-1α and OXPHOS upregulation [60]. These findings collectively nominate PGC-1α and Nrf2 as key HT targets, though precise regulatory crosstalk requires further dissection.

### 2.3. Modulation of Hydroxytyrosol on Mitophagy

Autophagy serves as a pivotal mechanism in sustaining cellular homeostasis and ensuring normal functionality through the metabolic processing and recycling of cellular components [61]. HT’s antioxidant and anti-inflammatory properties are closely linked to autophagy regulation, as extensively reviewed by Velotti et al. [62]. Mechanistically, HT upregulated p62 transcription via both sirtuin 1 (SIRT1)-dependent and -independent pathways, initiating cytoprotective autophagy in chondrocytes under oxidative stress [63]. Conversely, under inflammatory conditions, HT elevated the expression of autophagy-related proteins ATG5 and ATG7 while suppressing p62 through SIRT1-dependent signaling axis [64], demonstrating regulatory versatility of HT in autophagy. Our laboratory has also investigated HT’s autophagy regulatory mechanisms, revealing its capacity to induce autophagic impairment and apoptosis via ROS-dependent pathways, thereby suppressing the proliferation of human prostate cancer cells [65] and colon cancer cells [66]. Complementing these findings, we recently demonstrated that a novel cyclohexane-based HT derivative exhibited potent anti-proliferative efficacy against ovarian cancer cells through concurrent ROS amplification and autophagic flux inhibition [67]. These collective observations suggest that HT’s autophagy modulatory functions may present duality across normal versus malignant cellular environments.

Mitophagy is a selective autophagic process to eliminate impaired or non-functional mitochondria [68]. Reactive oxygen species (ROS) serve as crucial damage signals, inducing mtDNA damage, decreasing mitochondrial membrane potential, and promoting protein oxidation. Mitophagy represents a vital cellular response to curb the excessive generation of ROS [69]. Recent mechanistic investigations have demonstrated that HT safeguards against oxidative damage by activating the PI3K/Akt-Nrf2 signaling pathway and enhancing mitophagy. Mechanically, Nrf2 augments the expression of antioxidant enzymes, thereby neutralizing existing ROS, whereas mitophagy eliminates damaged mitochondria, thus diminishing ROS production [70]. Crucially, genetic inhibition of either Nrf2 or mitophagy abolished HT’s protective effects [70], underscoring the interdependence of these pathways. Complementary study by Dong and his team [35] identified HT-mediated AMPK/PINK1 pathway activation as a key driver of mitophagy, restoring mitochondrial cristae integrity and alleviating high-fat diet (HFD)-induced oxidative damage [35]. Collectively, these findings position HT as a multifunctional mediator of mitophagy-associated mitochondrial quality control mechanisms. It is noteworthy that there exists a close interplay between mitochondrial dynamics and mitophagy [71]. Although current research on the regulatory role of HT in mitochondrial dynamics during mitophagy remains limited, emerging evidence has demonstrated that HT modulates the expression of key mitochondrial fusion/fission proteins, including OPA1, Mfn2, and Drp1 [28,46,47]. Furthermore, study has revealed that HT enhances autophagic flux and facilitates autophagosome formation during autophagy activation [63]. Taken together, these findings suggest that HT may promote mitophagy progression through maintaining the equilibrium between mitochondrial fission and fusion processes and facilitating the formation of mitophagosomes. Future studies are imperative to delving deeper into the intricate mechanisms by which HT functions within the regulatory network governing mitochondrial dynamics and mitophagy. Notably, lifestyles may induce mitochondrial dysfunction through mechanisms such as triggering excessive production of ROS, impairments in mitochondrial biogenesis and mitophagy, as well as abnormalities in mitochondrial fusion and fission [72]. HT may serve as an “exercise mimetic” to offer a therapeutic strategy for lifestyle-related mitochondrial dysfunction and metabolic disorders by activating exercise-adapted pathways such as AMPK/PGC-1α-mediated mitochondrial biogenesis [73] and PINK1/Parkin-mediated mitophagy [74].

### 2.4. Modulation of Hydroxytyrosol on Mitochodnria-Endoplasmic Reticulum Crosstalk

The structural and functional synergy between mitochondria and the ER is essential for mitochondrial homeostasis and cellular physiology [75]. A recent study has shed light on the critical role played by the establishment of mitochondria–ER contact (MERC) sites in the regulation of mitophagy [76]. Yepuri et al. [77] underscored the crucial importance of Mfn2-facilitated mitochondria–ER interactions in governing mitochondrial turnover. Another investigation conducted by Li et al. [78] demonstrated mitochondrial Lon protease 1-preserved cardiac function by stabilizing MERC architecture and balancing fusion–fission dynamics. Accumulating evidence links disrupted mitochondria–ER interactions in both mitochondrial dysfunction and metabolic disease pathogenesis such as T2DM [79] and cardiovascular disorders [80,81].

Notably, emerging evidence position HT as a dual modulator of mitochondrial homeostasis and ER stress pathways. In vitro analyses revealed that HT alleviated tunicamycin-triggered unfolded protein response (UPR) in HepG2 cells by normalizing ER stress protein expression [82]. Parallel work demonstrated that HT’s antitumor effects [83] and neuroprotective effects [84] involved the modulation of ER calcium homeostasis. In line with these observations, recent in vivo studies by Xu et al. [85] and Wu et al. [86] reported that HT mitigated myocardial infarction by restoring both the mRNA and protein levels of ER stress markers GRP78 and CHOP. Consistently, a 10-week administration of HT has demonstrated efficacy in attenuating diet-induced chronic inflammation and insulin resistance in mice, achieved through the modulation of ER stress-related pathways [87]. Given HT’s concurrent regulation of Mfn2 (a MERC organizer) and ER stress effectors, we hypothesized its protective effects may stem from coordinated mitochondria–ER network modulation. Nevertheless, mechanistic details of this interorganelle regulation remain unresolved.

### 2.5. Modulation of Hydroxytyrosol on Gut Microbiota-Mitochondria Axis

The gut microbiota–mitochondria axis encompasses dynamic bidirectional communication between intestinal microbial communities and mitochondria. This interplay serves as a critical determinant of metabolic homeostasis, regulating systemic bioenergetics, immune modulation, and inflammatory cascades [88,89]. Accumulating evidence has highlighted that gut dysbiosis directly impaired mitochondrial quality control [90] and disrupted energy transduction efficiency [91,92]. Such perturbations propagate systemic metabolic dysregulation, driving pathologies spanning obesity [93], hepatic steatosis [94], malignancies [95], and neurodegenerative disorders [96], etc.

Recent investigations have demonstrated the modulatory capacity of HT towards gut microbiota. Song et al. [97] showed that HT could be metabolized by colonic microbiota into aryl hydrocarbon receptor-activating metabolites, which reinforce intestinal barrier integrity through microbiota restructuring. Complementary work by Han et al. [98] linked HT-driven microbiota shifts to altered plasma metabolite profiles. Preclinical evidence further revealed HT’s capacity to suppress pro-inflammatory microbial taxa while enriching short-chain fatty acid-producing bacteria, ameliorating murine colitis [99]. Miao et al. [100] extended these findings, showing HT alleviates ulcerative colitis via microbiota-dependent NLRP3 inflammasome inhibition. In metabolic contexts, Wang et al. [101] reported that HT reduced pathogenic bacterial populations, counteracting insulin resistance and metabolic dysregulation. Another study illuminated that HT reversed HFD-induced microbiota dysbiosis and intestinal barrier impairment, thus attenuating obesity-associated inflammation [30]. Notably, the beneficial attributes of HT could be transferred via fecal microbiota transplantation, underscoring that the salutary effects of HT are mediated through the modulation of gut microbiota [30]. Collectively, these findings implicate gut microbiota restructuring as a central mechanism of HT’s bioactivity. Considering the established microbiota–mitochondria crosstalk in metabolic homeostasis, HT may exert systemic benefits through this bidirectional axis, though mechanistic validation remains warranted.

**Figure 2:**
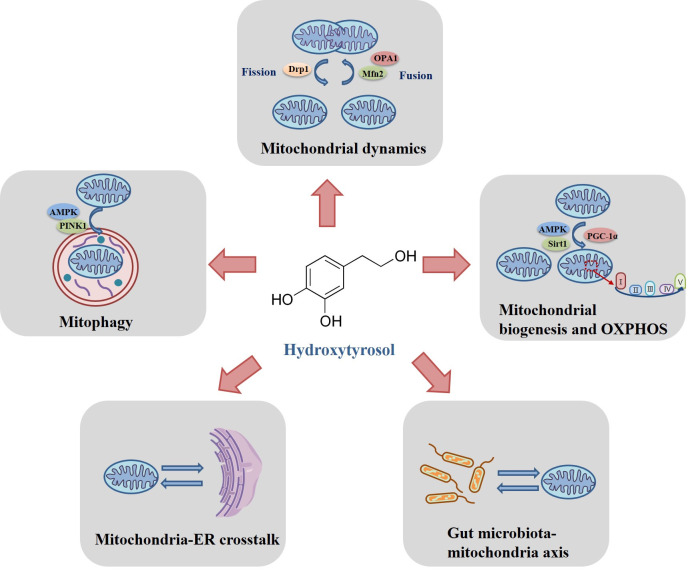
The regulatory role of hydroxytyrosol (HT) on mitochondrial homeostasis. Specifically, HT modulates mitochondrial dynamics (mainly via regulating fission-related protein Drp1 and fusion-related proteins Mfn2 and OPA1), mitochondrial biogenesis and OXPHOS (mainly via regulating AMPK, Sirt1 and PGC-1α), mitophagy (mainly via regulating AMPK and PINK1), mitochondria–ER interactions, and gut microbiota–mitochondria crosstalk.

The regulatory role of HT on the mitochondrial homeostasis networks is depicted in Figure 2.

## 3. The Protective Role of Hydroxytyrosol in Metabolic-Related Diseases

### 3.1. Type 2 Diabetes and Its Complications

Hyperglycemia is a fundamental pathological feature of MetS, primarily contributing to the decline of pancreatic β-cell function and the development of insulin resistance—two critical mechanisms in the pathogenesis of T2DM [102]. Our pioneering work first demonstrated HT as a MetS intervention, revealing its superior efficacy over metformin in mitigating hyperglycemia and insulin resistance in HFD-fed and db/db mice at 5% equivalent dosage [28]. Mechanistically, HT alleviated mitochondrial oxidative damage in hepatic and skeletal muscle tissues while restoring mitochondrial complex activity and Drp1-mediated fission dynamics [28]. Subsequent studies corroborated these findings, highlighting HT’s role in enhancing insulin sensitivity through mechanisms involving the regulation of endoplasmic reticulum stress [87] and modulation of gut microbiota [30]. A recent clinical trial further validated the efficacy of HT, showing that a 12-week dietary intervention rich in HT significantly decreased fasting blood glucose, insulin levels, and glycated hemoglobin in T2DM patients [31]. Emerging evidence also points to HT’s potential against T2DM-related complications, including diabetic cardiomyopathy [103], neuropathy [104], and retinopathy [105]. The benefits of HT on the aforementioned diabetic complications may be related to its antioxidant and anti-inflammatory properties. Moreover, recent studies have indicated that HT could inhibit the proliferation of vascular wall cells in diabetic rats by downregulating the expression of vascular cell adhesion molecule 1, as well as reducing oxidative stress and inflammatory responses, thereby improving diabetic vasculopathy [103]. However, a deeper understanding of the intricate regulatory mechanisms by which HT functions in diabetic complications, particularly those mechanisms associated with mitochondrial homeostasis, continues to require further elucidation. Collectively, these studies robustly support the therapeutic efficacy of HT in the prevention and management of T2DM and its complications.

### 3.2. Obesity, Hyperlipemia, and Nonalcoholic Fatty Liver

Dyslipidemia, marked by dysregulation in serum cholesterol, triglyceride levels, or both, is another significant pathological manifestation of MetS, elevating the risk for conditions such as obesity, hyperlipidemia, and NAFLD [106]. Recent evidence has revealed that HT could markedly ameliorate lipid metabolic disturbances by reducing dyslipidemia parameters such as triglycerides, total cholesterol, and LDL-cholesterol and reversing adipocyte hypertrophy. Mechanistically, these beneficial effects may be attributed to enhanced catalase and superoxide dismutase activities, bolstering the antioxidant defense system, and mitigating inflammatory responses [107]. Our previous studies have similarly demonstrated that HT not only benefits T2DM but also exhibits favorable effects in addressing HFD-induced obesity, hyperlipidemia, and NAFLD. Specifically, we observed that HT significantly reduced lipid droplet formation in both the liver and skeletal muscle of HFD-fed mice [28]. These effects are tightly associated with HT’s suppression of the SREBP1-FAS fatty acid synthesis pathway, indicating the regulatory role of HT on lipogenesis [28]. Our recent work has further elucidated that HT can enhance vascular endothelial health compromised by hyperlipidemia by promoting the nuclear translocation of Nrf2 and Foxo1, which are essential for maintaining redox balance and mitochondrial homeostasis [108]. Moreover, emerging evidence has highlighted HT’s positive impact on obesity-related complications, such as cognitive impairments linked to obesity, through anti-neuroinflammatory mechanisms [109], as well as the alleviation of obesity-related lymphedema [110]. Recent investigations have also reinforced HT’s advantageous role in dyslipidemia [107,111] and NAFLD [35,59,112,113,114]. HT exhibited significant effects in reducing lipid peroxidation, with various molecular mechanisms converging on its modulation of mitochondrial homeostasis [35,59,112,114]. However, it is important to note that a recent study using humanized mouse models indicated that excessive doses of HT could result in weight gain and metabolic issues [115], emphasizing the need to consider species differences and the careful use of HT dosages in clinical settings to avoid potential adverse effects.

### 3.3. Hypertension-Related Diseases

Alongside hyperglycemia and hyperlipidemia, hypertension is a crucial pathological component of MetS. Recent studies have revealed the therapeutic potential of HT in managing hypertension and its associated complications. Menichini et al. [116] observed that a compound formulation containing HT effectively reduced blood pressure and vascular reactivity in pregnant hypertensive mice, likely through the modulation of endothelial nitric oxide synthase (eNOS). Further research indicated that HT provided cardioprotective effects by regulating mitochondrial homeostasis and enhancing the function of the electron transport chain [117]. Human clinical trials have shown promising results, with studies reporting that a six-week regimen of olive extract rich in HT significantly lowered both systolic and diastolic blood pressure, as well as blood lipid levels, in prehypertensive patients [118]. Additionally, HT-rich olive extract markedly improved endothelial function in hypertensive individuals [119]. Research by Iakovidis et al. [120] suggested that short-term supplementation with olive oil extract high in HT correlated with trends toward improved left ventricular diastolic function and aortic elasticity in patients with chronic coronary artery disease. Despite these promising outcomes, the specific regulatory mechanisms through which HT exerts its beneficial effects on hypertension and related disorders, particularly regarding mitochondrial homeostasis, require further exploration.

## 4. Challenges and Future Perspectives

### 4.1. Direct Targets of Hydroxytyrosol: Awaiting Further Elucidation

Despite substantial progress in characterizing HT-mediated mitochondrial homeostasis and its therapeutic potential in metabolic disorders, critical knowledge gaps hinder its clinical translation. Unlike synthetic drugs targeting single pathways, HT exerts pleiotropic effects by modulating mitochondrial-related networks, which coordinates multi-organ metabolic adaptation. This aligns with plant metabolites’ evolutionary role in stress resistance [121], underscoring their therapeutic potential for complex diseases like MetS. A primary challenge resides in delineating HT’s direct molecular targets within mitochondrial regulatory networks. While current studies implicate HT’s modulation of mitochondrial homeostasis-related proteins (such as PGC-1α, OPA1, AMPK, PINK1, Sirt1, and Nrf2), these effects could reflect secondary adaptations rather than primary target engagement. Current research has not yet elucidated whether there are direct interactions between HT and the aforementioned proteins. Notably, HT demonstrates hormetic (biphasic dose-response) effects analogous to other plant-derived polyphenols: At physiological concentrations, HT elicits beneficial effects via activation of the AMPK/SIRT1/PGC-1α-mediated mitochondrial biogenesis pathway [58,59], whereas supraphysiological doses may perturb intracellular redox homeostasis, potentially exerting cytotoxic effects [109]. These hermetic effects emphasize the careful use of HT dosages in clinical settings to avoid potential adverse effects. Advanced chemoproteomic strategies—such as activity-based protein profiling (ABPP) [122] or thermal proteome profiling (TPP) [123]—are urgently needed to map HT’s high-affinity molecular interactors. Furthermore, the mechanistic interplay between HT and mitochondria-organelle crosstalk remains particularly obscure. Resolving whether HT directly modulates MERC site dynamics or indirectly influences interorganelle communication via redox/nutrient-sensing pathways requires advanced imaging techniques (e.g., cryo-electron tomography) [124] coupled with organelle-specific, multi-omics analysis. Similarly, HT’s regulation of the gut microbiota–mitochondria axis demands multi-omics integration (such as metagenomics, metabolomics, and mitochondrial proteomics) to disentangle host–microbe interactions from direct mitochondrial effects. In addition, the variations in the molecular targets of HT among diverse cell types, including normal cells and tumorigenic cells, still necessitates thorough clarification and further investigation.

### 4.2. Mitochondrial Delivery System for Enhanced Targeting

A critical limitation in current research on HT lies in its nonspecific subcellular distribution, which significantly reduces therapeutic efficacy by diluting its action across cellular compartments. The development of mitochondrial delivery systems represents a paradigm-shifting strategy to overcome this bottleneck. Engineered nanocarriers, such as triphenylphosphine-conjugated nanoparticles [125] and mitochondria-targeting liposomes [126], are capable of traversing both cellular and mitochondrial membranes while preserving the stability of drugs, thus becoming an effective strategy for mitochondrial drug delivery especially in antitumor application. Our recent study has revealed that triphenylphosphine-conjugated HT demonstrated superior anti-inflammatory efficacy and enhanced therapeutic efficacy in mitigating hyperlipidemia-associated mitochondrial dysfunction and endothelial damage compared to unmodified HT, providing compelling evidence for the effectiveness of mitochondrial-targeted drug delivery systems in therapeutic applications [108].

The clinical translation of HT is further constrained by its inherent chemical instability, as rapid oxidation at physiological pH and susceptibility to enzymatic degradation drastically reduce its bioavailability. To address this limitation, our laboratory has pioneered the development of stabilized HT analogs through rational structural modifications, including HT acetate [21,36,127], HT butyrate [128], 2-(3,4-Dihydroxyphenyl)ethyl 3-hydroxybutanoate [129], and cyclohexane-HT derivative [67]. These HT derivatives have demonstrated superior bioactivity and therapeutic potential in multiple pathologies [36,67,127,128,129]. Future endeavors may consider the integration of these HT derivatives with mitochondrial delivery systems as a strategy to dually augment the therapeutic efficacy of HT.

### 4.3. Synergistic Therapies for Boosting Efficacy

The potential of HT to act synergistically with complementary therapeutic agents presents promising opportunities for enhancing its therapeutic efficacy in metabolic disorders. Our preliminary investigation demonstrated that a formulated antioxidant complex containing HT, coenzyme Q10, α-lipoic acid, and acetyl-L-carnitine exhibited mitochondrial-enhancing synergy [130]. This multicomponent regimen effectively attenuated disuse-induced muscle atrophy in a rodent hindlimb suspension model [130]. Emerging evidence from other research teams further substantiates the therapeutic benefits of HT-containing combinations, including cardioprotective effects through co-administration with oleuropein and oleocanthal [34], as well as gastrointestinal protection [131] and NAFLD mitigation [132] when combined with vitamin E. Nevertheless, identifying optimal therapeutic combinations and deciphering the molecular basis of these synergistic interactions remain significant challenges in translational research. Strategic investigation should prioritize systematic evaluation of diverse combination therapies through integrative approaches incorporating computational pharmacology and mechanistically validated preclinical models. Furthermore, detailed characterization of pharmacokinetic compatibility and pharmacodynamic interplay between HT and co-administered compounds will be essential for developing optimized therapeutic regimens that maximize clinical benefits while maintaining favorable safety profiles.

## 5. Conclusions

In summary, contemporary evidence from our investigations and existing studies establishes HT as a multifaceted mitochondrial modulator that transcends its classical role as an antioxidant agent. HT demonstrates remarkable capacity to orchestrate mitochondrial homeostasis through regulation of dynamic remodeling processes, bioenergetic optimization, organelle turnover mechanisms, and inter-organellar communication networks—particularly along the mitochondria–ER axis and gut microbiota crosstalk. These coordinated actions of HT collectively contribute to the amelioration of MetS and its associated conditions, such as T2DM and its complications, obesity, hyperlipidemia, NAFLD, and hypertension-related disorders. Future investigations should prioritize elucidating HT’s precise molecular targets and effector pathways, engineering mitochondria-specific delivery platforms, and exploring HT’s synergistic potential with existing therapeutics. Addressing these critical challenges will be essential for translating HT’s mitochondrial regulatory properties into clinically viable interventions for complex metabolic diseases.

## Figures and Tables

**Table 1:** The biologic effect of hydroxytyrosol.

	Model	Experimental Outcome	Ref.
Antioxidant	MPP+-Induced Striatal Lipid Peroxidation in Rats model	HT increased GSH activity and GSH/GSSG ratio	[37]
Anti-viral	Peripheral blood mononuclear cells isolated from healthy blood donors	Hydroxytyrosol inhibited HIV-1 infections in cell	[38]
Antibacterial	Spectrum Beta-Lactamases	HT disrupted bacterial enzymes crucial for maintaining cell integrity and DNA replication	[39]
Anti-cancer	breast cancer cell lines (MDA-MB-231, MDA-MB-468, and SUM159) colorectal cancer cell line (Caco-2)	HT modulated intracellular copper levels inhibited tumor progression HT increased Caco-2 cell DNA methylation, decreased EDNRA expression	[40] [41]

MPP+: 1-methyl−4-phenylpyridinium, GSH: Glutathione, GSSG: glutathione disulfide, EDNRA: endothelin receptor type A gene.

## Abbreviations

The following abbreviations are used in this manuscript:

HT	Hydroxytyrosol
MetS	Metabolic Syndrome
T2DM	Type 2 Diabetes Mellitus
NAFLD	Non-Alcoholic Fatty Liver Disease
OXPHOS	Oxidative Phosphorylation
ER	Endoplasmic Reticulum
OPA1	Optic Atrophy 1
Drp1	Dynamin-Related Protein 1
Mfn2	Mitofusin 2
PGC-1α	Peroxisome Proliferator-Activated Receptor γ Coactivator 1-α
mtDNA	Mitochondrial DNA
SIRT1	Sirtuin 1
ROS	Reactive Oxygen Species
HFD	High-Fat Diet
MERC	Mitochondria–ER Contact
UPR	Unfolded Protein Response
eNOS	Endothelial Nitric Oxide Synthase
ABPP	Activity-Based Protein Profiling
TPP	Thermal Proteome Profiling
